# Bridging the gap in outpatient care for adolescent eating disorders: Usability of a digital mental health intervention for anorexia or bulimia nervosa

**DOI:** 10.3389/fpsyt.2025.1640889

**Published:** 2025-09-25

**Authors:** Szarah Sanchez Roman, Emily Panek, Larissa Niemeyer, Victor Saase, Matthias Norden, Marie Ottilie Frenkel

**Affiliations:** ^1^ Institute of Sport and Sport Sciences, Heidelberg University, Heidelberg, Germany; ^2^ Arbeitseinheit Affektive Neuropsychologie, Abteilung Psychologie, Fakultät für Psychologie und Sportwissenschaften, Universität Bielefeld, Bielefeld, Germany; ^3^ Center for Cognitive Interaction Technology Bielefeld University, Bielefeld University, Bielefeld, Germany; ^4^ Faculty Health, Medical and Life Sciences, Psychology in Health Care, Furtwangen University, Freiburg, Germany

**Keywords:** anorexia nervosa (AN), bulimia nervosa (BN), eating disorders, mHealth, user centered design, adolescents, digital intervention, mental health

## Abstract

Eating disorders such as Anorexia Nervosa (AN) and Bulimia Nervosa (BN) are serious mental illnesses that typically emerge during adolescence and often become chronic. In Germany, affected individuals wait an average of 26 weeks for outpatient psychotherapy, creating a critical treatment gap. Digital interventions may serve as a bridging solution, particularly for the digitally oriented younger population. This study evaluated the Usability, Acceptance and perceived Usefulness of a cognitive behavioral therapy (CBT)-based mobile intervention for adolescents with AN or BN. Data collection occurred in two phases: an initial pilot with 10 mentally healthy adolescents (mean age = 13.8, SD = 1.2; n_female = 7), followed by a second phase with 20 adolescents (mean age = 14.9, SD = 1.6; n_female = 20) diagnosed with an eating disorder. Assessments included the German Mobile Health App Usability Questionnaire (G-MAUQ) and semi-structured focus group interviews. Qualitative data was analyzed using content analysis according to Kuckartz. On a 1–7 scale, the clinical group and the healthy group reported similar mean usability scores (M = 5.97, SD = 0.44 vs. M = 5.84, SD = 0.44), indicating high usability in both groups. Feedback clustered around four themes: Interface Satisfaction, Feature Acceptance, Ease of Use, and Usefulness. Personalization through companions, gamification, and design were well received. The meal planner was particularly valued for its practical relevance. Focus group interviews highlighted both strengths (e.g., personal approach, interactive format, structured meal planning) and areas for improvement (e.g., text length). Given its scalability, this CBT-based intervention may help fill existing service gaps in the healthcare of adolescents with eating disorders and complement existing treatment pathways.

## 1 Introduction

One to three percent of adolescents living in the European Union suffered from a clinically relevant eating disorder (ED) before onset of the COVID-19 pandemic (1, 2). During the pandemic, this number increased enormously and was also connected with higher rates of hospitalization (3). With an estimated 100,000 children and adolescents currently meeting the diagnostic criteria of an ED in accordance with ICD-10 in Germany, an increase in incidence was observed in the period from 2019 to 2021 (4). Especially Anorexia Nervosa (AN) remains the deadliest psychiatric disorder in childhood and adolescence, through either suicide or the long-term consequences of severe underweight (5, 6). It also often has a chronic course with an average disease duration of six years (7). Untreated ED often lead to prolonged and costly inpatient treatment in the long term, whereas early diagnosis and interventions improve recovery outcomes and reduce the overall burden on healthcare systems (8).

However, timely interventions for an effective therapy are often prevented by average waiting times of 6 months for outpatient therapy, due to insufficient healthcare resources in Germany (9, 10). Contributing factors are, the urban-rural divide, long travel times of over 45 minutes in one direction, and numerous vacant specialist positions in the outpatient sector (11). To date, 20% of child and adolescent psychiatrists are over 60 years old and will retire in the coming years (12), which will likely exacerbate the already strained care situation. On the other hand, child and adolescent psychiatry is also suffering from difficulties in recruiting new professionals. In summary, there are recurring therapy gaps during the patient journey that promote the progression and chronicity of the disorders.

In the future, digital technologies hold immense potential to enhance the accessibility and efficiency of healthcare systems. Innovative treatment approaches, such as digital mobile health interventions (DMHIs), can therefore represent an attractive and necessary complement to already established therapies (13). For the group of particularly media-savvy Digital Natives, a therapy app can be a desirable option. According to the HealthApps4Teens Report 2021, 97% of all 12- to 19-year-olds in Germany owned a smartphone (14), and 84% have been searching for health information online (15). According to a survey, 70% of participants aged 16 to 25 reported having installed an app to promote their mental health during COVID-19 (16).

Currently, there is still no DMHI in Germany specifically developed for children and adolescents suffering from anorexia nervosa, Bulimia Nervosa (BN) or its atypical forms. For this reason, eatappie, a mental health smartphone application for children and adolescents aged 12 to 17 years with eating disorders, is being developed. It provides therapy content, exercises and tools according to established medical guidelines, adapted for the digital domain and self-guided use. It offers psychoeducation, instructions for behavioral change and interactive exercises based on cognitive behavioral psychotherapy as well as meal planning and tracking. By this the app is aiming at counteracting the disease progression in patients suffering from anorexia and bulimia in the transition periods when no face-to-face therapy is available. Complementary to family-based treatment, individual psychotherapy is the first-line treatment approach for eating disorders and there is growing evidence that CBT is an effective therapy option for children and adolescents (17, 18). Furthermore, digital CBT interventions were shown to be effective in adult population suffering from BN (19). One challenge in the development of such an app is the competition with the large number of entertainment apps, which are specifically designed to keep users engaged for as long as possible (20). One quarter of the installed mental health apps is never used, while another quarter are uninstalled already after a single session (21). This highlights the importance of high Usability standards (22) and the early and active involvement of patients in the development process (23).

To the best of our knowledge, this is the first work introducing a smartphone-based intervention designed for untreated and/or waiting list children and adolescents diagnosed with AN, BN or its atypical forms in Germany. Firstly, the prototype of this novel app named eatappie is presented and its design and concept based on existing treatment options like CBT are explained. Secondly, an Usability study also investigating Acceptance and Usefulness including mentally healthy and children and adolescents diagnosed with AN or BN is described. Finally, the potentials of eatappie for bridging the gap between diagnosis and in-person psychotherapeutic care is discussed and future studies are outlined.

## 2 Methods

In this section, first the concept and design of the eatappie application is described explaining the methodological background. This is then followed by the methodological details of the Usability evaluation.

### 2.1 Prototype development

The app eatappie was developed as a medical device by three of the authors (SSR; LN; VS) with the flutter framework, supporting native deployment to iOS and Android devices. Data is stored locally on the user’s device; continuous internet access is not required for use. The app uses state of the art two-factor authentication and encryption. The patients’ personal data is locally encrypted on the device and sent for backup to a server in Germany. The key for decryption is derived from the patients’ password, which is not stored on the server. Only the email address of the user is available on the server for authentication. Together these measures ensure confidentiality of patients’ medical data even in cases of a data breach of the server, a takeover of the users’ email account or password leakage. The content of the app is largely based on the recommendation of the German guideline „Diagnostik und Behandlung der Essstörungen “ (24) and is initially released in German language only. The app contains evidence-based CBT modules and interactive exercises supported by a meal planner with protocol function. The composition and order of the unit topics have been specifically developed for eatappie and do not exist in this form elsewhere. The exercises have been newly designed for digital, interactive use, whereas some established and well-tested methods (e.g. skills for emotion regulation, behavioral analysis for binge eating or psychoeducational content) have been adapted for the digital format. The combination of different elements aims to engage the particularly media-savvy group of digital natives in ongoing participation (25). A screen recording providing an overview of the app’s structure is included in the **Supplementary Material**.

#### 2.1.1 Features

##### 2.1.1.1 Therapeutic modules with exercises

Each phase (psychoeducation, modification of behavior, stabilization) is composed of four modules. Furthermore, three optional modules regarding the topics “binge eating”, “feelings and emotions” and “perfectionism” are available. A new module is unlocked each week. The content of the module itself is divided into several therapy sessions. Each therapy lesson is supported by a practical interactive exercise to consolidate the learned knowledge. The pace for the completion of each module is tailored to the user, resulting in a processing period which can range from minimum 12 weeks to several months (see also **Table 1** in the supplement).

**Table 1:** Content of the 12-weeks smartphone application intervention (eatappie) designed for AN and BN.

Phase 1: Psychoeducation	
1 *Introduction*	Get to know the functioning of the app; introducing the therapy companion; information about therapy forms and BMI;
2 *Psychoeducation*	General information about eating disorders; physical
3 *individual disease*	consequences; sexuality and puberty; motivational aspects
* Model*	Recognizing individual causes and triggers of eating disorders
4 *Nutrition*	Information about balanced diet according to DGE, portion sizes, introduction to meal planner

Phase 2: Modification of behavior	
5 *Motivation*	Stages of change, motivational content
6 *Emotion Regulation*	Relaxation training, emotion regulation skills, skills addressing the urge to move
7 *Cognitive restructuring*	Recognizing, analyzing and changing automatic negative thoughts
8 *Forbidden food*	Identifying forbidden foods, mindful eating and behavioral experiments

Phase 3: Stabilization	
9 *Self-esteem and resources*	Training self-confidence and acceptance, recognizing personal resources, healthy sport
10 *Life despite illness*	Goal setting, social environment, practicing to go grocery shopping
11 *Social media*	Dealing with ideals of beauty representation, threatful content and recovery accounts
12 *Relapse prevention*	Relapse prevention strategies, “first-aid-kit” for risk situations

Optional content	
1 *Feelings*	Information about basic emotions, training to recognize and assign them
2 *Binge-eating episodes*	Analysis of trigger situations, strategies to avoid binge eating behavior, “emergency plan”
3 *Perfectionism*	Recognize and change perfectionistic beliefs

##### 2.1.1.2 Meal planner and protocol function

**Figure 1:**
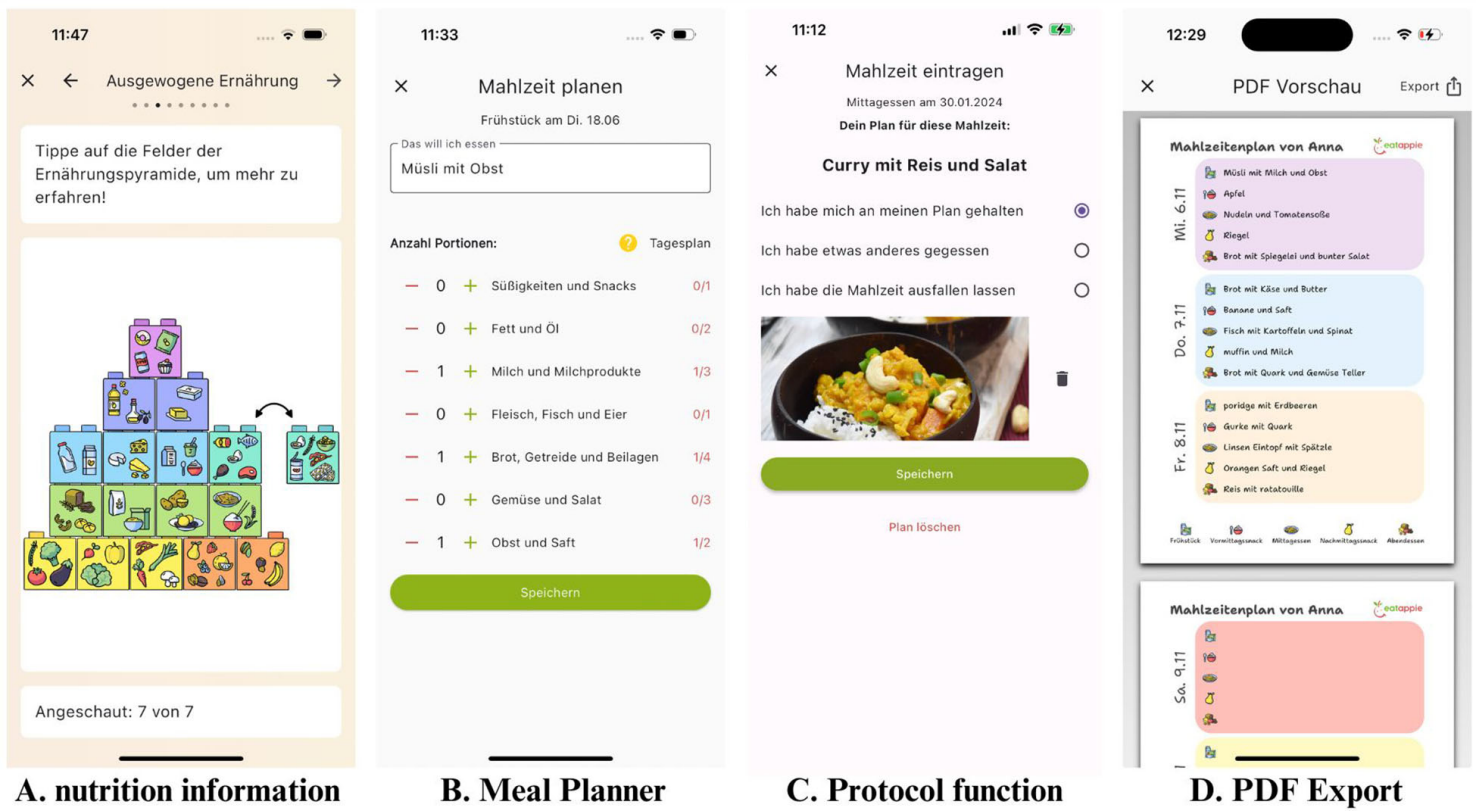
Design and features of the meal planner. Picture **(A)** shows psychoeducational content regarding balanced nutrition. Picture **(B)** shows the exampalry planning of a meal with proportions of food groups. Picture **(C)** shows the protocol function with optional photo upload. Picture **(D)** shows the examplary PDF Export of meal plan for 3 days, that can be send to a physician or therapist. Design made by Birgit Jansen.

In parallel, users can plan up to five meals per day and subsequently log them. Meals can be entered as free text, and users can specify food groups and portion sizes instead of using calorie counts. This approach aims to minimize disease-related calorie counting and guide users toward a more intuitive approach to nutrition (see also **Figure 1**).

#### 2.1.2 Gamification

##### 2.1.2.1 Virtual therapy-companion

**Figure 2:**
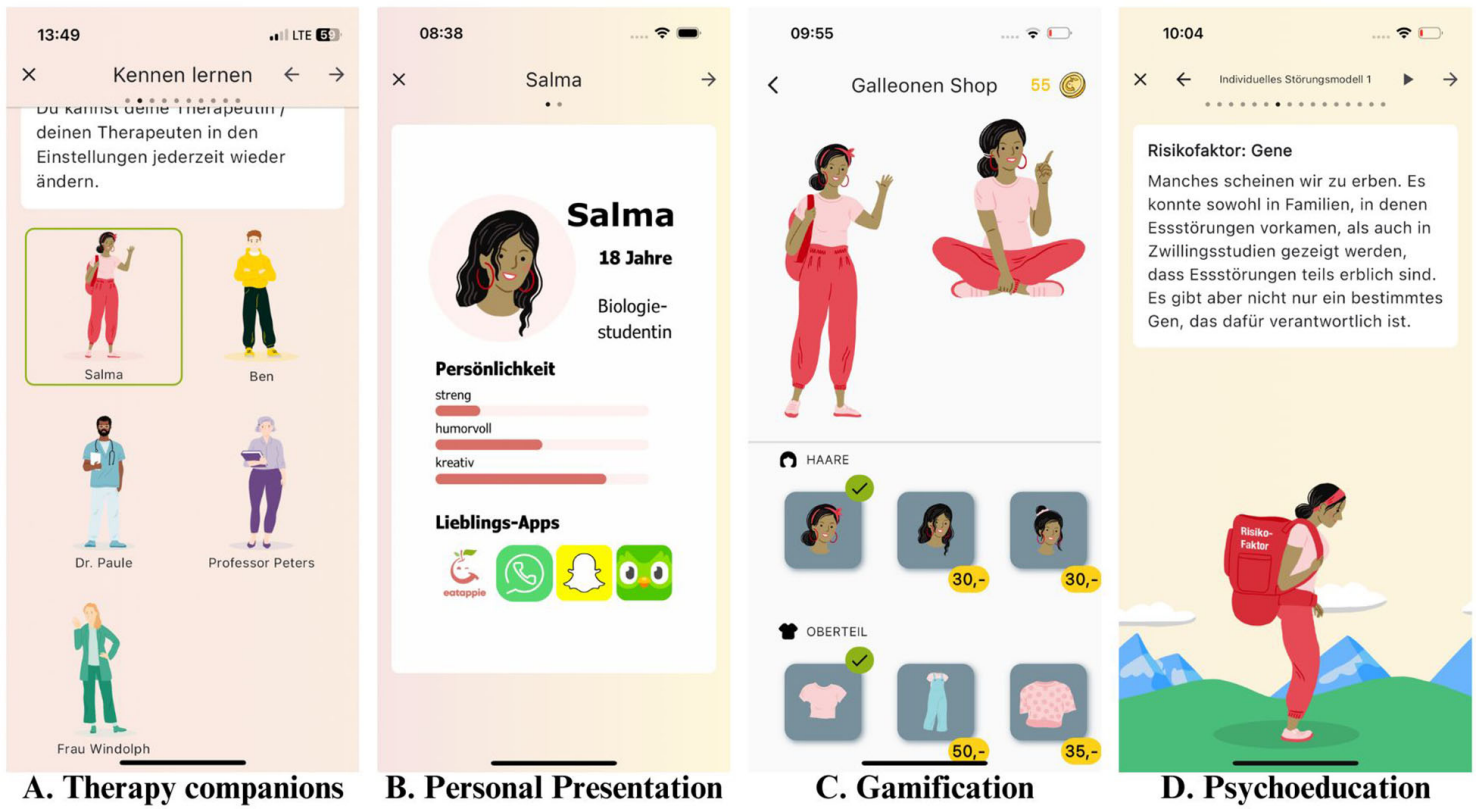
Design and features of the application prototype. Picture **(A)** shows the introduction to the therapy companions. Picture **(B)** shows the exampalry in detail presentation of the character "Salma". Picture **(C)** shows the accessories shop used as part of the behavioral reinforcement model. Picture **(D)** shows one of the psychoeducational contents. Design made by Birgit Jansen.

Users can choose between five different therapy-companions (three therapists, two young adults formerly suffering from an eating disorder). The companion leads in the app, motivates and gives instructions. This approach aims to mimic a therapeutic relationship(see also **Figure 2**).

##### 2.1.2.2 Achievements

Upon achieving various daily or weekly goals (e.g., “Planning 20 meals for the week,”), users earn badges. In addition, users can earn “eatappie Galleons” for their correct answers in quizzes and for completing exercises. These can be exchanged in the integrated shop within the app for accessories for the virtual therapy companions (see also **Figure 2**). For completing particularly important therapy sections like completion of the psychoeducation phase, the user can earn certificates that should increase their motivation and provide valuable feedback to clinicians and therapists regarding how well the individuals are engaging and how informed they are about their condition. This gamification approach aims to reinforce desired behaviors and to encourage ongoing participation amongst children and adolescents.

### 2.2 Usability evaluation

Participants and recruitment: For evaluating the Usability, Acceptance and Usefulness we recruited two groups, starting with a group of 10 mentally healthy children and adolescents aged from 12–15 years (M = 13.8, SD = 1.2) from a local school. The second group consisted of 20 patients, aged 12–17 years (M = 14.9, SD = 1.6), suffering from eating disorders according to ICD-10 criteria recruited through multiple channels, including child and adolescent psychiatric clinics (inpatient and outpatient) and a pediatric-focused clinical nutritionist. Healthcare professionals received detailed information about the study protocol and were invited to refer eligible participants to the study. The sample size corresponds to the guidelines typically recommended for qualitative research (26). We aimed to have a divers, representative patient population including male participants and a broad spectrum of eating disorders (AN, BN, atypical forms). Patients with suicidal thoughts, major depressive episodes, or a BMI below the third percentile were excluded as the capability for outpatient treatment cannot be assumed. Enrolment was conducted from March to November 2024. Participants and their legal guardians (e.g., parents) were informed verbally and in written form about the procedure and provided their written consent. The Ethics Committee of the faculty of behavioral and empirical cultural sciences at Heidelberg University approved the study (AZ Ples 2024 1/2).

#### 2.2.1 Procedure

Qualitative data was collected with the goal to deeper understand the user’s experience. This was achieved through semi-structured focus group interviews lasting an average of about 120 minutes. In three cases, individual interviews were conducted, as the respective participants were unable to attend group sessions due to their health conditions or scheduling conflicts. All interviews were conducted by a resident in child and adolescent psychiatry (SSR or LN) and were digitally recorded and transcribed later. Smartphones with a preinstalled app version were distributed. Both software systems IOS and Android were tested. The first part of the focus group interview consisted of a 20 min individual testing time, during which the participants were asked to explore the app on their own. This was followed by 10 tasks in the app to test comprehension of specific functions of eatappie (e.g. registration, create a new password, modify consent for data usage, find and work on a specific therapy module). Then, a dialog was initiated and moderated using questions previously defined in the focus group interview guide. Finally, the participants completed two questionnaires to evaluate the user experience and record sociodemographic characteristics such as age, diagnosis, smartphone ownership, and average screen time.

#### 2.2.2 Usability questionnaire

User experience was assessed by the mobile Health App Usability Questionnaire (G-MAUQ (27);). Eighteen items (seven-point likert scale, ranging from 1 to 7 (don´t agree at all - fully agree, with higher numbers indicating greater agreement); content validity S-CVI = 0.93) evaluate three subscales (Ease of Use, Interface Satisfaction and Usefulness). The G-MAUQ has shown a high correlation with the System Usability Scale (SUS; r = 0.77, p < 0.001), one of the most widely used Questionnaires in Usability research. Although the original validation of the G-MAUQ does not define explicit cut-off values, score interpretation was aligned with Usability benchmarks adapted from the SUS (28).

Specifically, mean scores ≥6.0 were considered to indicate very high Usability, whereas scores between 5.0 and 5.9 indicates good Usability, scores between 4.0 and 4.9 to indicated acceptable Usability, and scores below 4.0 to suggested a need for improvement. This categorization provides a practical framework for interpreting the Usability of mobile health applications in adolescent populations. As the study primarily sought to explore Usability and User Perceptions, descriptive statistics were deemed sufficient and no inferential analyses were undertaken.

In accordance with the questionnaire developer’s guidelines, missing values were imputed using the neutral midpoint value of 4 (i.e., neither agree nor disagree (29);.

#### 2.2.3 Qualitative data

For a better understanding of the requirements for a digital psychotherapeutic intervention for children and adolescents with eating disorders, and to evaluate how individuals in this age group perceive the presented solution and potentially adapt to it, a semi-structured focus group interview guide was developed. The guide contains 31 questions, which can be categorized into 4 main thematic areas: 1. Ease of Use, 2. Acceptability and Attractiveness, 3. Usefulness and 4. Proposals for modifications and enhancements to the intervention. All focus group interviews were conducted in German. Further in-depth questions were added when deemed appropriate.

#### 2.2.4 Data analysis

The focus group interviews were recorded with a dictaphone and subsequently transcribed verbatim. The data collected during the focus groups interviews were thematically analyzed using MAXQDA (version 24.4.1.). In the first stage of the analysis, each transcript was read multiple times without applying any coding in order to fully familiarize ourselves with the content. This initial stage followed the approach of qualitative content analysis as outlined by Kuckartz (30), ensuring that no premature thematization occurred. In the next step, four overarching categories were defined in alignment with the primary research objectives. The transcripts were then systematically coded based on these categories. The process of continuous thematization was applied to ensure that the data aligned with these predefined groups. Subsequently, subcategories were derived inductively from recurring themes within the data, allowing for a more granular understanding of the content. The coding process was conducted by EP, and the developed codes were then jointly reviewed and discussed by EP and SSR to ensure inter-rater reliability and consistency in interpretation.

## 3 Results

### 3.1 Participants

The group of healthy individuals comprised 10 individuals (70% female) with a mean age of 13.8 years (SD = 1.2, **Table 2**). All individuals of the group suffering from an ED (n = 20) were all female, with a mean age of 14.9 years (SD = 1.6) and diagnosed with AN.

**Table 2:** Sample characteristics.

Characteritics	Healthy population (n = 10)	Affected population (n = 20)
Age in years, M (SD)	13.8 (1.2)	14.9 (1.6)
gender, f (%)	70	100
F50.0 diagnose (%)	-	100
Total screen time, Min (SD)	327 (115.3)	163 (62.5)
- thereof social media	120.8 (82.39)	73.8 (49.2)
- thereof chat/phone	75.5 (57.8)	43.6 (26.5)
- thereof gaming	46.5 (63.9)	10.5 (26.7)
- thereof watching videos	38 (48.7)	20.5 (33.3)
- thereof other (photos, music, Netflix)	47 (60.7)	17.9 (36)

All of them owned smartphones since an average age of 10.53 years (SD = 1.1). The group of healthy adolescents reported a mean total daily screen time of 327 minutes (SD = 115.3), whereas adolescents diagnosed with AN reported an average of 163 minutes (SD = 62.5). In both groups, social media use constituted the largest share of screen time, followed by communication via messaging applications (see also **Table 2**).

### 3.2 Quantitative data

The Usability and Usefulness of the eatappie prototype was assessed using the German version of the Mobile App Usability Questionnaire (G-MAUQ). Across all participants, five individuals (two healthy adolescents and three adolescents with lived experience) missed one to two item responses, resulting in a total of eight imputations (1.5% of all responses). The healthy group reported a mean overall Usability score of M = 5.84, SD = 0.44, while the clinical group of adolescents with diagnosed eating disorders reported a minimally higher mean score of M = 5.97, SD = 0.44. Values exceeded the threshold of 5.0 for good Usability. According to SUS related thresholds, subscale analyses revealed good ratings for Ease of Use and Interface Satisfaction in the healthy group, (M = 5.84, SD = 0.27; M = 5.98, SD = 0.38) and very high Usability ratings for both subscales in the clinical group (M = 6.18, SD = 0.15; M = 6.03, SD = 0.48). The Usefulness subscale was only administered to the clinical sample, as the healthy participants were not suited to evaluate the therapeutic relevance of the content. In this group, the Usefulness subscale yielded a mean score of M = 5.74, SD = 0.52, corresponding to a good level of perceived utility. (see also **Table 3**).

**Table 3:** Descriptive statistics at scale level of the G-MAUQ.

Scale level of the G-MAUQ	Healthy population (n = 10) M (SD)	Affected population (n = 20) M (SD)
G-MAUQ_ease of use	5.84 (0.27)	6.18 (0.15)
G-MAUQ_interface satisfaction	5.98 (0.38)	6.03 (0.48)
G-MAUQ_usefulness	-	5.74 (0.52)
G-MAUQ_total	5.84 (0.44)	5.97 (0.44)

### 3.3 Qualitative data

Interview duration ranged from 12 minutes 40 seconds to 61 minutes 31 seconds. The four main categories emerging from the focus group interviews are 1) Interface Satisfaction, 2) Ease of Use, 3) Acceptance of Features and 4) Usefulness. A total of 440 data units were identified that contained relevant statements which could be assigned to the categories. Of these, 80 data units fell under the category Interface Satisfaction, and 48 under Ease of Use. The largest share was accounted for by statements relating to Acceptance of Features, making up 198 of all data units. Statements concerning Usefulness were found in 45 units. For each subcategory, both evaluations regarding the current version of the app, as well as suggestions to improve possible deficiencies, are included in the analysis. One additional category (Suggestions and Impulses) was defined to address further proposals and critical aspects that could not be assigned to the four main categories. Additional Suggestions and Impulses were contained in 35 data units. **Table 4** shows example quotes of the respondents for each subcategory.

**Table 4:** Categories, code groups, example quotes*.

Interface Satisfaction (n = 80)	
Design and colors (n = 25)	*„Almost like a safe place, really. Because everything was so beautifully designed. (14 years)* *I liked the colors, but I think it would be cool if you could change the colors, for example, to make it more personal for everyone.”* (15 years)
Images and graphic elements (n = 16)	„*The ratio of images to text was actually good. I also liked the interactive pictures. (14 years)* *I actually find factual texts better without pictures, because then they’re not so distracting. Because the pictures are always a bit distracting. “* (15 years)
Language (n = 13)	*„Everything was well explained and easy to understand. And also relatively carefully phrased, so that you’re not bombarded with ‘how did you get that [the eating disorder]? So it doesn’t make you feel guilty. “* (17 years)
Texts (n = 17)	*„So for me personally, it was actually too much text. If you have a bit of difficulty with attention and such. “* (15 years) *„So the texts, which were long, were also packed with a lot of information and so on. So I wanted to keep reading because I was interested. That’s why the texts didn’t seem so long once you’d started reading. “* (13 years)
Audio files (n = 9)	*„Yes, I find it very practical. I’ve used it a lot because sometimes I’m a bit too lazy to read. Then it’s quite practical. “* (14 years) *„I can always remember things better when I read them. “* (13 years) *„I think it’s good. Maybe, if it wasn’t too much effort, [it’d be nice] to integrate a male voice. “*(13 years)
Ease of Use (n = 48)	
Navigation and menu position (n = 26)	*„I found it simple and understandable*. “ (14 years) *„And I also found it very easy to use in general. I found my way around the app relatively quickly. “* (15 years) *„It took me a very long time to enter the weight.”* (17 years)
Login and passcodes (n = 22)	*„The only thing I found a bit difficult at the beginning was the unlock code, because I was a bit confused about password and unlock code.”* (15 years) *„I definitely preferred [the secret words] over having to additionally log in via email.”* (15 years) *„Because whenever you enter your email address you also get email notifications and then you always have so many emails and have to delete them again. So I think it’s actually really cool with the code.”* (14 years)
Acceptance of Features (n = 198)	
Motivation system “eatappie Galleons” (n = 27)	*„So I think this is above all an incentive to keep working on the app.”* (15 years) *„And then you also have a goal to work towards.”* (14 years)
Streak-feature (n = 14)	*„It would motivate me, but it would also stress me out, because then I’d be under pressure to enter a meal, otherwise I’d lose the medal again. But I actually think it’s a good idea.”* (15 years) *„It’s a bit like that, because you put yourself under a bit of pressure because you don’t want to lose the streak. and actually the galleons are enough as a reward system.”* (14 years)
Therapy companions (n = 49)	*„I also thought it was cool that you could choose your own therapist. Because I read through them all and I chose the one that I thought suited me best in terms of appearance and so on. And I thought it was cool that you could choose just like a real person.”* (13 years) *„I also liked the fact that they all looked different and even human. So they didn’t all have the same figure, weren’t standing the same way, wearing the same clothes or I don’t know what. I thought that was also very good.”* (17 years) *„It really felt like you were writing to a person who was there for you.”* (15 years)
Shop (n = 35)	*“I would’ve liked a few more choices [of items in the shop].”* (12 years) *„Because there was only one animal, [I’d like it] if you could choose more pets or if there were more hairstyles.”* (13 years).
Quizzes and tasks (n = 46)	*„I found them [quizzes] helpful because they repeated what I’d just read, so that it was stored in my brain in a way.”* (13 years) *„I actually liked it because it added a bit of variety – not just theory, so to speak.”* (15 years)
Name and logo (n = 27)	*„I think it’s a very cool name, because it fits, it’s also about food. And it sounds good because I always find that when an app sounds so hard you don’t want to have it on your mobile phone.”* (13 years) *„And this could also be one of those [recipe] apps. So you can’t directly see what it’s about.”* (13 years)
Usefulness (n = 45)	
Meal planner (n = 20)	*„In any case [I could imagine] using the meal plan. I actually think it’s good that you can download it.”* (14 years) *„I think it’s easier [to use the meal plan] on my cell phone. It’s also that feeling of shame again, if you always carry the meal plan around with you, it’s weird to take it out during meals.”* (15 years) *„I found some of the information in the meal plan too unclear. I would have liked a few more examples.”* (15 years)
Providing information (n = 12)	*„Hmm, I already knew most of the things, but I think repeating it again is useful.”* (14 years) *„What stuck with me the most were these skills [tools or coping strategies]. I just thought it was really cool that you could tick off what you’re currently experiencing, how intense it is, what you need right now and so on, because I think those are really the moments where most people struggle — in those acute situations*,
	*[especially] not giving in to the urge to move [i.e., the compulsive need to exercise]. So I thought that was really good.”* (15 years) *„And it was also very important that there was not only a medical perspective but also a psychological one.”* (17 years)
Consideration as addition for therapy (n = 7)	*„So I think that [the skill] would help me a lot, because you often don’t have a direct contact person at home or something. So that would definitely help me and all these info texts in general, so that I keep reminding myself of them or something.”* (15 years) *„[If I got warning alerts in the app], I would tell my parents, my doctors or therapists … Yes, I would take it seriously.”* (13 years) *„So you have the feeling that it’s not an obligation, that you can do it voluntarily for yourself. I think that’s really great. And also a bit of playfulness, yes, I think that’s great.”* (14 years)
Fulfilment of expectations (n = 6)	*„Actually [I expected] exactly what it is. Simply that it helps you. Yes, it really is like therapy. Just like when you go to outpatient therapy, only that you can do it digitally.”* (14 years) *„I did think that maybe there would be something about weight. And otherwise I expected the app to assess what stage I’m currently at or whether I really need a lot of help. Maybe tell me that I should go to a day clinic or maybe an inpatient stay.”* (13 years)
Further suggestions (n = 35)	*„What I’d also like is if you could write down your hobbies – and have the motivational sayings relate to them.”* (12 years) *„[I’d like it if] you could enter a sports plan. Because exercise is also important in general. But so that it’s not too much.”* (14 years) *„Maybe also something about self-esteem or body image, because most people’s body image isn’t quite correct either.”* (15 years)

*All example statements were provided by female participants from the clinical adolescent group.

#### 3.3.1 Interface satisfaction

##### 3.3.1.1 Design & colors

Participants found the app’s abstract and minimalist design modern and appropriate for the target group. The bright, diverse color scheme was perceived as joyful and motivating.

However, greater customization was suggested to increase satisfaction and avoid a gender-biased appearance.

##### 3.3.1.2 Images and graphic elements

Overall, participants liked the implementation of images in addition to the text elements, to loosen up the text-based elements and sustain engagement. They described them as visually appealing and found the overall image–text balance appropriate, though some suggested adding more visuals. Interactive images and graphics, such as those in the nutrition pyramid, were particularly appreciated. Participants also valued the use of animations, as they added a dynamic element to the app. However, they felt the current frequency was sufficient, as excessive movement could be distracting and irritating.

##### 3.3.1.3 Language

Participants found the language age-appropriate and clear. All textual elements, including dialogues and quizzes, were seen as suitable for the target group. The tone was perceived as natural and personally engaging.

Users valued the sensitive phrasing regarding potential feelings of embarrassment or guilt in affected users. Gender-inclusive language was well received and posed no comprehension issues.

##### 3.3.1.4 Texts

Concerning the amount of text, participants held varied opinions. Some participants felt the amount was excessive and potentially demotivating or boring.

Others considered it appropriate, noting the importance of retaining key information that could not be effectively condensed further.

To reduce the overwhelming character of long text passages, participants suggesting to include short summaries at the end of a text block or to make bullet points alternatively. In addition, shorter text segments or alternatively, more modular formats, like speech bubbles, were appreciated to make the texts more convenient to read and understand.

##### 3.3.1.5 Audio files

Audio files were considered a useful, though optional, alternative to text.

Many participants preferred reading, noting that key information is more reliably conveyed through text. For users less inclined to read, audio reduced effort and helped overcome usage barriers. The recordings were described as clear and easy to understand. Suggestions included customizable playback speed, a male voice option, and resuming audio from the last time stamp instead of starting again.

#### 3.3.2 Ease of use

##### 3.3.2.1 Navigation and menu position

In general, the app was described as simple to navigate. Overall, the app was perceived as intuitive and self-explanatory.

Some participants experienced initial difficulties navigating the app, particularly in locating specific settings. The weight-setting feature caused confusion, as it was expected in a separate menu rather than under “general settings.”

##### 3.3.2.2 Login and passwords

The most frequently mentioned issue with the app was initial confusion with the passwords and “secret words”. Due to the sensitivity of the stored data, a three-factor authentication was implemented for the initial app login, consisting of a self-chosen password, six predefined “secret words,” and the user’s email address. For subsequent logins, entering the password alone is sufficient for authentication. Participants reported that they did not understand which of the passwords to use. In addition, some expressed concerns about memorizing the passcodes.

They overall agreed and understood that the procedure is appropriate for a health app due to security aspects.

#### 3.3.3 Acceptability/attractiveness of features

##### 3.3.3.1 Motivation system

The gamification approach was seen as positive and motivational, though not essential. Participants appreciated the motivation system with coins called “galleons”. Opinions on the Streak-Feature were controversial. While no participant rejected the idea outright, some expressed concerns about potential stress and pressure from daily app usage. Others worried that excessive focus on maintaining the streak (a feature that rewards consecutive daily use) could overshadow the app’s primary purpose.

##### 3.3.3.2 Therapy companions

Participants evaluated the companions as very positive and useful, with no negative opinions. Users appreciated the individual choice of companions based on their preferences and the ability to change them at any time. The diverse personalities, personal information, professions, and therapeutic approaches of the companions were highlighted as positive aspects. Participants also valued the relatable backstories, which made them feel understood, especially when facing similar difficulties.

Interactive conversations with the companions were seen as a valid alternative to text passages, feeling more individual and adapted to the user. The variety in body types, styles, and behaviors of the companions was appreciated. Respondents suggested including more male characters to better engage male users and increasing the frequency of companion appearances.

##### 3.3.3.3 Shop

All participants under the age of 14 who provided a statement on this topic expressed a positive opinion about the shop, stating that it increased motivation and enjoyment. Participants aged 15 to 17 generally felt that the shop was not essential but considered it as a positive additional feature. Participants generally found it motivating for engagement with the app, particularly due to the customizable and individual nature of the companions and overall app usage. While the feature was appreciated, suggestions included expanding the variety of options, such as clothing, hairstyles, pets, and environmental or room accessories.

##### 3.3.3.4 Quizzes and tasks

The quizzes were in general perceived as useful to strengthen the previously learned information. In particular, the interactive approach was evaluated as precious and important. Participants reported that they especially liked the complex tasks where they actively had to write, pull and draw elements instead of just ticking boxes.

Some healthy participants indicated that the instructions could be more concise and clearly structured. Additionally, they requested more examples and enhanced feedback to better clarify the purpose of the quizzes. These features were modified in response to feedback and were no longer mentioned in later rounds of testing.

##### 3.3.3.5 Name and logo

The app-name was perceived positively, and the participants found it creative and easy to remember. As intended, the name eatappie was associated with nutrition and happiness. None of them found the name inconvenient or embarrassing given its subtlety and because it could also be the name for a non-therapeutical app. The respondents also highlighted positively that although the logo is associated with the content, it does not explicitly indicate that the app is for therapy. This makes it more comfortable for them if a non-affected peer happens to see the app on their screen.

#### 3.3.4 Usefulness

##### 3.3.4.1 Meal planner

When asked which element was particularly useful and memorable, participants most frequently mentioned the meal planner, appreciating its utility and applicability to their daily lives. All participants found the meal planner easy to use, valuing its design and straightforward input method. However, some reported confusion regarding portion size selection and suggested including more examples and detailed input methods. They also requested greater flexibility in timing/planning and stronger adaptation to individual needs.

The export function was perceived as highly useful and a key benefit of the meal planner. Most respondents preferred the digital meal planner over the paper version, citing its accessibility, availability via smartphone, and subtlety in public use.

##### 3.3.4.2 Providing information

Participants appreciated the balance between medical and psychological content in the app, reporting that they learned valuable information applicable to their lives and therapy. They found that the app stimulated reflection and encouraged behavior change, particularly regarding unhealthy habits. While some content was already known to participants, especially those familiar with therapy, they still found it useful for reinforcement or while waiting for therapy. The most memorable elements were the nutrition pyramid, physical consequences, the skills overview and the collection of possible problem-solving-strategies in emotionally stressful situations, due to their strong real-life applicability.

##### 3.3.4.3 Consideration as addition for therapy

Overall, the app was perceived as a valuable supplement to therapy. Participants indicated they could envision sharing the meal planner and protocols with their nutritionists or therapists and discussing their learnings with parents. Participants affirmed the app’s potential as an adjunct to ambulant therapy and believed their therapists would approve its use.

They also liked that the app was voluntary and gives a feeling of autonomy and independence, if the patients have difficulties to implement the initial therapeutic interventions to their real life. While not associated with pressure or fear, participants still regarded it as a serious tool rather than mere entertainment.

##### 3.3.4.4 Fulfilment of expectations

When asked about their expectations for a health app, the majority of participants agreed that the app “already hit the goal.” Specifically, they expected the meal planner and protocol function to be typical elements of therapy for eating disorder.

Some participants mentioned that they had expected and would also consider it very useful if the app would provide addresses/emergency contacts or therapeutic institutions in the users’ region.

Regarding the purpose of the app, it was expected that the app motivates the user and elicits a positive and optimistic feeling. It was further mentioned that the app is regarded as a long-term helping tool and not as an immediate solution that helps after a single usage.

## 4 Discussion

### 4.1 Principal findings

The present study evaluated the Usability, Acceptance, and Usefulness of a CBT-based mobile intervention designed for adolescents with AN and BN, but tested here only in AN population. Overall, both healthy and affected users reported good Usability scores on the German version of the mHealth App Usability Questionnaire (G-MAUQ), with affected participants rating the app minimally better. These values are indicative of good Usability according to the benchmarks defined earlier.

To our knowledge, this is the first time the German version of the questionnaire has been used in an adolescent population. The G-MAUQ is a relatively new instrument specifically developed for evaluating DMHIs. Although the developers have not yet established cut-off values (29), previous studies have demonstrated a high correlation with the well-established System Usability Scale (SUS (27);).

By adapting the Usability assessment, the present study aims to facilitate comparability with existing literature. In their evaluation of the Care Me physical self-care app, Rezaee and colleagues reported a total mean Usability score of 6.28 on the Persian version of the MAUQ among 15 Iranian adolescents aged 13 to 15, indicating a very high level of Usability (31). In contrast, previous studies using the G-MAUQ in adult populations have yielded comparatively lower scores. For instance, Tacke and colleagues reported a mean score of 4.50 (SD = 1.40) in a sample of 53 adults evaluating the Herzfit app, a cardiovascular mHealth intervention (32). These differences may reflect higher levels of digital literacy and greater openness to digital interventions among younger users. This interpretation aligns with findings from a Chinese study, which identified significantly higher engagement with mHealth apps among young female college students (33).

While the quantitative data revealed no differences between the two study phases at a descriptive level, qualitative feedback disclosed divergent user needs: healthy participants preferred less text and more audiovisual content, whereas affected users responded positively to the simplified and revised content. These findings may indicate a successful adaptation process but could also reflect social desirability or heightened treatment expectations among the affected group. Gender may have influenced the results, given that only female participants were included in the clinical group.

Overall, participants appreciated the app’s modern, minimalistic design and uplifting color scheme, although some suggested customizable color options to enhance personalization. The language was perceived as clear, engaging, and inclusive. Visual and auditory elements such as images, audio files, and animations were valued for improving engagement. Preferences for interactivity and personalization emerged as central themes across user feedback, consistent with findings from previous Usability studies on ED apps (21, 34).

The qualitative data further indicated that participants generally found the app intuitive, although some initially experienced confusion with the menu layout and the weight-setting feature. The login process was viewed as secure and appropriate for a medical product; however, users recommended clearer instructions and fewer secret words during registration.

Adolescents expressed interest in additional features such as direct access to weight curves or information on exercise and sports in the context of eating disorders; however, these features were intentionally excluded by the development team due to concerns about their therapeutic appropriateness. There is a risk that including such content may inadvertently reinforce or maintain disordered behaviors, despite potentially improving Usability. These findings highlight a central tension in digital health design: balancing user satisfaction and accessibility with clinical integrity and safety. This underscores the importance of evaluating user feedback through a clinical lens and involving experienced therapists throughout the development process.

The gamification system, particularly the “galleons” reward system, was perceived as motivating, especially by younger participants. They found it helped with goal-orientation and engagement, and the “galleons” provided direct positive feedback, keeping them motivated. However, opinions on the Streak feature were mixed, with some users reporting feelings of pressure. This concern has also been documented in previous studies (21) and emphasizes the need to implement reward systems with caution.

The therapy companions were appreciated for their personalization and diversity, though users requested more male characters and more frequent interactions. In general, participants felt that the app met their expectations. They valued features such as the meal planner, skills overview, and problem-solving strategies as useful additions to ongoing therapy. The balance of medical and psychological content was positively received, and optional modules related to body image, sports, and motivational content were suggested. These findings suggest that the app may offer meaningful therapeutic support in outpatient care contexts.

Although none of the adolescents in this study explicitly expressed concerns regarding screen time, this issue is frequently discussed in the broader scientific discourse on mHealth. Critics argue that such apps may contribute to increased digital exposure—especially in populations already vulnerable to excessive media use. To address this concern, the app was deliberately designed to enable effective daily use within approximately 15 minutes.

Interestingly, the affected adolescent group in this study reported notably lower screen times compared to their healthy peers. This finding may be influenced by gender distribution, social desirability, or illness-related behavioral patterns that are not yet fully understood. This observation warrants further investigation in larger and more diverse samples.

Finally, existing literature suggests that shame can serve as a barrier to the adoption of digital interventions in populations with eating disorders (35). In this study, the app’s name and visual branding were perceived as neutral, which may have helped reduce this barrier. However, this aspect should be further explored in non-treatment-seeking populations, as all participants in the current sample were already attending in-person psychotherapy.

### 4.2 Strengths and limitations

This study provides initial evidence that a CBT-based mobile application for adolescents with AN is both usable and well accepted by its intended target group. Participants especially valued personalization, gamification, interactive elements, and the digital meal planner—highlighting the importance of tailoring digital mental health interventions to the developmental needs of younger users.

Notably, affected adolescents rated Usability slightly higher than healthy controls, suggesting high receptiveness among those with lived experience. The app’s alignment with adolescent preferences may reflect the influence of youth-informed development, though further research is needed to determine its impact on adherence.

The present study has several limitations. First, the sample included only female adolescents with AN, already receiving treatment, limiting generalizability to untreated individuals, males, or adolescents with BN. Treatment experience may have biased perceptions of app Usability and Usefulness positively. Second, the absence of participants with BN restricts conclusions about the app’s applicability across the full spectrum of ED. Although both diagnostic groups were originally targeted, recruitment challenges led to a final sample of AN only. Third, cross-sectional design focused on Usability, Acceptability and Usefulness, preventing conclusions on long-term effectiveness, sustained engagement, or clinical outcomes. Future longitudinal research should assess impacts on symptoms, adherence, and functional recovery in real-world. A randomized controlled trial (RCT) is currently planned. Finally, the qualitative insights are limited by a small homogenous sample and specific recruitment context. Larger, more diverse samples, spanning different genders, treatment status, and cultural backgrounds, are needed to validate and extend these findings.

## 5 Conclusions

This study suggests that a CBT-based mobile app tailored for adolescents with AN is usable and well received. Features such as personalization, gamification, interactive content, and the digital meal planner were rated particularly favorably.

Given the critical shortage of timely care options and long waiting times for outpatient treatment, digital tools such as eatappie may help fill existing service gaps in the healthcare of eating disorders, offering therapeutic support before, during, or after in-person therapy.

These findings highlight the potential of youth-centered digital interventions to complement existing treatment pathways effectively.

## Data Availability

The raw data supporting the conclusions of this article will be made available by the authors, without undue reservation.
